# Flexible Pressure Sensor with Micro-Structure Arrays Based on PDMS and PEDOT:PSS/PUD&CNTs Composite Film with 3D Printing

**DOI:** 10.3390/ma14216499

**Published:** 2021-10-29

**Authors:** Yiwei Shao, Qi Zhang, Yulong Zhao, Xing Pang, Mingjie Liu, Dongliang Zhang, Xiaoya Liang

**Affiliations:** State Key Laboratory for Manufacturing Systems Engineering, School of Mechanical Engineering, Xi’an Jiaotong University, Xi’an 710049, China; imporeed@stu.xjtu.edu.cn (Y.S.); px2014@stu.xjtu.edu.cn (X.P.); liumingjie@stu.xjtu.edu.cn (M.L.); zhangdl666@stu.xjtu.edu.cn (D.Z.); liang_xiaoya@stu.xjtu.edu.cn (X.L.)

**Keywords:** flexible pressure sensor, microstructure, 3D printing, composite film

## Abstract

Flexible pressure sensors are widely used in different fields, especially in human motion, robot monitoring and medical treatment. Herein, a flexible pressure sensor consists of the flat top plate, and the microstructured bottom plate is developed. Both plates are made of polydimethylsiloxane (PDMS) by molding from the 3D printed template. The contact surfaces of the top and bottom plates are coated with a mixture of poly (3,4-ethylenedioxythiophene) poly (styrene sulfonate) (PEDOT:PSS) and polyurethane dispersion (PUD) as stretchable film electrodes with carbon nanotubes on the electrode surface. By employing 3D printing technology, using digital light processing (DLP), the fabrication of the sensor is low-cost and fast. The sensor models with different microstructures are first analyzed by the Finite Element Method (FEM), and then the models are fabricated and tested. The sensor with 5 × 5 hemispheres has a sensitivity of 3.54 × 10^−3^ S/kPa in the range of 0–22.2 kPa. The zero-temperature coefficient is −0.0064%FS/°C. The durability test is carried out for 2000 cycles, and it remains stable during the whole test. This work represents progress in flexible pressure sensing and demonstrates the advantages of 3D printing technology in sensor processing.

## 1. Introduction

Due to the wide application of flexible pressure sensors in human motion [1,2,3], robot monitoring [4,5] and medical treatment [6,7,8,9], research on flexible pressure sensors has become popular in the past decade. Such sensors with high flexibility can convert pressure information input into an electrical signal output. Among them, capacitive and piezoresistive sensors are mostly reported. Capacitive sensors usually have two electrodes, sandwiching a dielectric layer. When pressure is applied, the distance between the electrodes changes, the dielectric constant of the dielectric layer changes or the electrode area changes [1,9,10,11,12,13,14,15,16,17,18,19,20,21,22,23,24,25]. By using different dielectric materials, changing the electrode size, etc., capacitive sensors meeting different requirements can be designed. However, due to the limitation of the size of electrodes, the capacitance is small, and the stray capacitance is relatively large, which is susceptible to external interference and affects the measurement accuracy.

The piezoresistive sensor has a simple structure, and the circuit design is easy [6]. This kind of sensor usually deforms under pressure and detects the pressure by the resistance change in three different ways: (1) The piezoresistive characteristics of the material itself [2,4,26]; (2) Doped carbon nanotubes, graphene, silver nanowires and other conductive fillers form more electrical contacts or a tunneling effect [3,5,27,28,29,30,31,32]; (3) The contact resistance of the contact area between the two electrodes changes [6,8,33,34,35,36,37]. For the third type of piezoresistive sensors, the common way to improve performance is to add fine microstructures onto the electrode contact surface. Compared with a device without microstructures [32], when an external pressure is applied within a certain range, the microstructure increases the change in the contact area between the two electrodes, improving the sensitivity of the sensor. By adjusting the shape and size of the microstructure, different performance sensors can be easily obtained. Micro pyramids are the most common microstructure in pressure sensors [6,33,34,36]. As reported by Choong’s work in 2014, a piezoresistive pressure sensor was achieved by coating a compressible substrate containing an array of micro pyramids, which was obtained by replication of silicon process templates, and had a sensitivity of 4.88 kPa^−1^ over a wide range of pressures from 0.37 to 5.9 kPa [6]. Moreover, microcylindrical and other structures were also reported and fabricated in a similar method [22,35,37].

Although the performance of these sensors is excellent and the silicon process is mature and precise, the fabrication process has its disadvantages. The silicon process is not only time-consuming and costly but can also generally only process planar structures. Complex surface structures, such as curved surfaces, are hard to process. It is necessary to study other fabrication processes, such as three-dimensional (3D) printing technologies [38]. Three-dimensional printing can directly generate parts of any shape from computer graphics data without machining, thereby greatly shortening the product development cycle and reducing costs. Moreover, 3D printing can also print some complicated appearances, such as curved surfaces and inclined surfaces, which are hard to fabricate by traditional silicon processes, expanding more ideas to design novel sensors.

Here, by 3D printing, the reasonable design and manufacturing method of a flexible pressure sensor is proposed. FEM is applied to simulate the stress distribution and resistance of sensors with different microstructures and density distributions. The results show that the sensor performance is dependent on the shape of microstructures, and the hemisphere is found to be a proper geometric structure that achieves the best sensor performance in terms of sensitivity and measurement range compared to pyramid and cone, while sensors with different hemisphere density distributions have similar responses but different measurement ranges. To simplify the manufacturing process, the templates with microstructures are fabricated by 3D printing. By molding the templates with microstructures, PDMS bottom plates with microstructures are created, and flat top plates without microstructures are obtained in the same way. Then, a thin layer of PEDOT:PSS and PUD mixture is evenly applied to the contact surface of the top and bottom plates and CNTs are distributed on the top of this layer of film electrode. Finally, the flat top plate and microstructured bottom plate are assembled to form the pressure before testing.

## 2. Materials and Methods

### 2.1. Sensor Design

**Figure 1:**
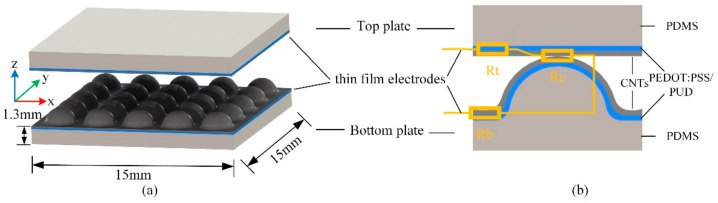
The proposed pressure sensor: (**a**) basic structure; (**b**) simplified sensor model.

The schematic diagram of the proposed flexible pressure sensors is shown in Figure 1a. The sensor was composed of upper and lower parts. The main body of the two parts was a flat square plate with a thickness of 1.3 mm and a side length of 15.0 mm. The surface of the lower part was evenly distributed with micro-structure protrusions, while the upper part was a completely flat plate.

**Figure 2:**
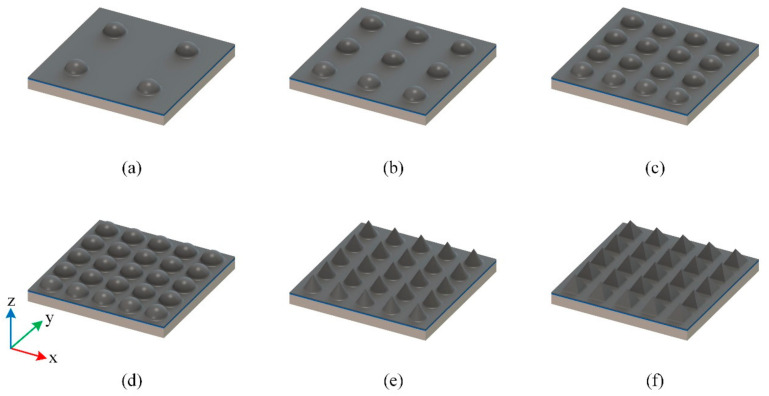
Schematic diagram of the designed sensors with 6 different structures: (**a**) 2 × 2 hemispheres; (**b**) 3 × 3 hemispheres; (**c**) 4 × 4 hemispheres; (**d**) 5 × 5 hemispheres; (**e**) 5 × 5 cones; (**f**) 5 × 5 pyramids.

Here we designed 6 different structures for the lower part: 2 × 2 hemispheres, 3 × 3 hemispheres, 4 × 4 hemispheres, 5 × 5 hemispheres with a diameter of 2.0 mm, 5 × 5 cones with a diameter of 2.0 mm on the bottom and 5 × 5 pyramids with a side length of 2.0 mm on the bottom, as shown in Figure 2.

To analyze the change trend of resistance under pressure, the sensor model was simplified [6], as shown in Figure 1b. The total resistance (*R*) consists of top plate resistance (*R_t_*), contact resistance (*R_c_*) and bottom plate resistance (*R_b_*):

(1) $$R=R_{t}+R_{c}+R_{b}$$

When the pressure is applied, the change of resistance could be described:

(2) $$\Delta R=R-R_{0}=\left(R_{t}+R_{c}+R_{b}\right)-\left(R_{t0}+R_{c0}+R_{b0}\right)$$

Since the top plate resistance was almost invariable, Equation (2) could be simplified to Equation (3):

(3) $$\Delta R\approx \left(R_{c}-R_{c0}\right)+\left(R_{b}-R_{b0}\right)$$

According to Ohm’s law,

(4) $$R=\frac{\rho L}{A}$$

where *L* was the length of the resistor, *A* was the cross-sectional area and *ρ* was the resistivity.

Equation (3) was rewritten to Equation (5):

(5) $$\Delta R=\rho_{c}\left(\frac{L_{c}}{A_{c}}\right.-\left.\frac{L_{\mathrm{c0}}}{A_{\mathrm{c0}}}\right)+\rho_{b}\left(\frac{L_{b}}{D_{b}C_{b}}\right.-\left.\frac{L_{\mathrm{b0}}}{D_{\mathrm{b0}}C_{\mathrm{b0}}}\right)$$

where *A_c_* was the contact area, *L_c_* was the film electrodes thickness at the contact area, *D_b_* was the film thickness at the side of micro-structure, *L_b_* was the film length at the side of micro-structure and *C_b_* was the perimeter of the contact area.

When the pressure was applied, *L_c_* and *L_b_* decreased, and *A_c_*, *D_b_* and *C_b_* increased, resulting in the resistance of the sensor decreasing.

### 2.2. Sensor Simulation

To analyze the influence of microstructure protrusions of different shapes and sizes on the performance of the sensor, the finite element method was introduced. The software used for simulation was COMSOL 5.6. Six sensor models with the different microstructures mentioned above were imported into the software, both top and bottom plates were modeled by hyperelasticity and the material model was the Mooney–Rivlin model, two parameters, with C_10_ 2.4 × 10^5^ Pa, C_01_ 6.6 × 10^4^ Pa and bulk modulus κ 1.25 × 10^7^ Pa. Then external displacement was applied, pushing the top plate downward to make contacts with the microstructures on the bottom plate. The pressure distance increased from 0 to 0.6 mm, and the change curve between displacement and force and resistance was obtained. The specific results were as follows.

**Figure 3:**
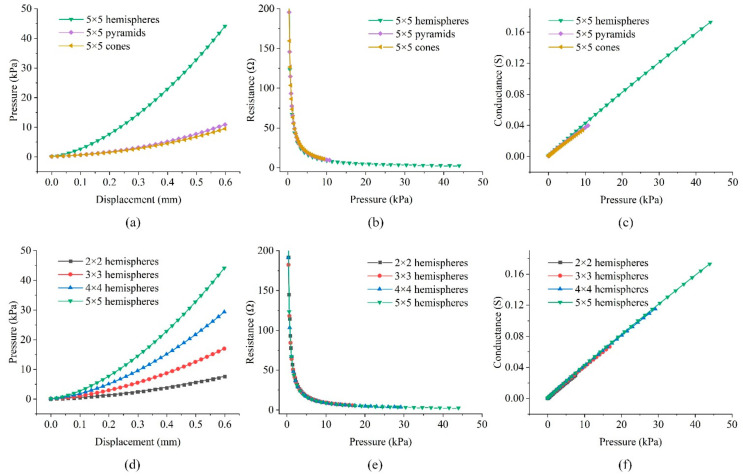
FEM results of the sensors with different microstructures: (**a**–**c**) Sensors with 5 × 5 hemispheres, pyramids and cones; (**d**–**f**) Sensors with different density distribution of hemispheres.

In the simulation results of the pressure change with the displacement of the depression, the protrusions of different structures were compared. As shown in Figure 3a, when the number of protrusions was 5 × 5, different structures had different pressure curves. In the same depression displacement of 0.6 mm, the sensor with hemispherical structures had the largest range of 44.09 kPa, while the sensor with pyramidal structures and the conical structures had a similar range, which is less than a quarter of that of the sensor with hemispherical structures.

Next, we studied the hemispheric protrusions with different distribution densities on the bottom plate surface of the same size. As shown in Figure 3d, it was obvious that as the number of hemispheres increases, the pressure also increases. Analysis of the data showed that the pressure was proportional to the number of hemispheres, and each hemisphere provided a pressure of approximately 1.76 kPa when the pressure displacement was 0.6 mm. This was also in line with common sense, and the sensor can be regarded as a parallel connection of different numbers of single hemispherical structure sensors. In this way, the higher the number of hemispheres, the larger the measurement range of the sensor.

Then the change of the sensor’s resistance with pressure was studied. As shown in Figure 3b,e, 6 types of sensors with different types and numbers of protrusions were compared. Although the structure and number of protrusions cause a huge difference in range, the resistance-pressure curves of all sensors in the same range were very similar. In the range of 0–5 kPa, the resistance rapidly decreased from the maximum value to 20 Ω. When the pressure was higher than 5 kPa, the resistance decreased very slowly. The inverse of the resistance was used to get the conductance. As shown in Figure 3c,f, the conductance changed almost linearly with pressure. The conductance-pressure curves of 5 × 5 hemispheres, pyramids and cones were relatively similar, while the conductance-pressure curves of sensors with different numbers of hemisphere protrusions almost overlap. No matter how the number of hemispheres changed, the output resistance of the sensor remained unchanged under pressure. Therefore, we could design sensors with different hemispherical density distributions according to the different ranges required by the measurement requirements, without the need for major modifications to the subsequent circuit.

**Figure 4:**
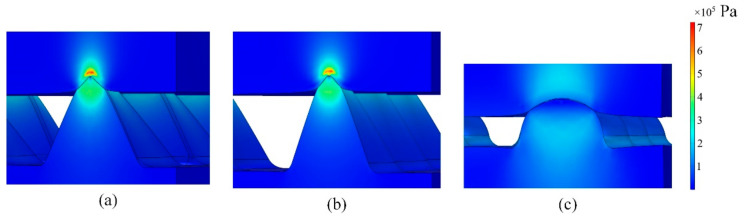
Von Mises stress of (**a**) pyramid, (**b**) cone and (**c**) hemisphere structures under 0.6 mm of displacement.

Meanwhile, the stress of the sensors with pyramid and cone structures was concentrated on the top of the microstructure, where the top plate and the microstructures on the bottom plate were in contact. The stress of the sensor with a hemispherical structure was relatively dispersed, and the maximum stress was much smaller than that of the sensor with pyramid and cone structures. When the applied displacement was 0.6 mm, the maximum von Mises stress of pyramid, cone and hemisphere structures were 6.40 × 10^5^, 7.24 × 10^5^ and 2.15 × 10^5^ Pa, respectively, as shown in Figure 4. Larger stress would cause the conductive film to be easily damaged. 

### 2.3. Sensor Fabrication

**Figure 5:**
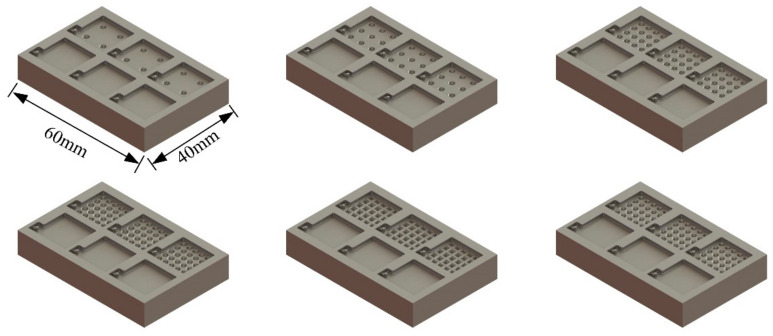
Designed templates.

The templates with micro-structure were designed, as shown in Figure 5 below.

The templates were divided into two parts, one part was the top plate, and the other was the bottom plate with different structure depressions. High-temperature-resistant resin (High Temp, Formlabs, Somerville, MA, USA) was used for processing the template with a 3D Digital Light Processing (DLP) printer (M-Jewelry U50, MAKEX, Ningbo, China). This printer is a desktop 3D printer with a lateral resolution of 50 µm and a thickness resolution of 5 µm. The high-temperature resin offers a heat deflection temperature (HDT) of 238 °C at 0.45 MPa. During printing, the template is printed from bottom to top in a vertical orientation.

**Figure 6:**
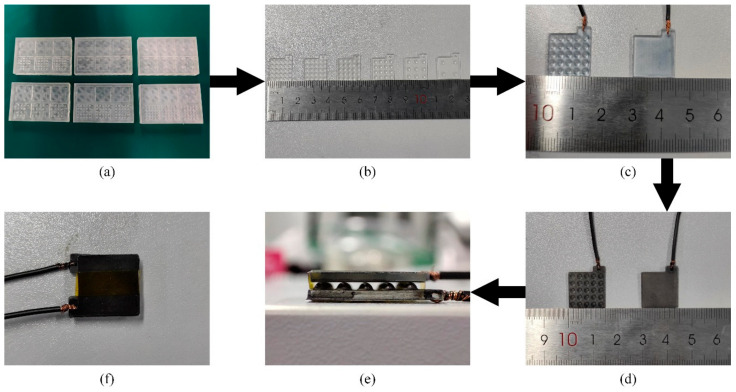
The fabrication process of sensors: (**a**) Template printing; (**b**) Template after cleaning and curing; (**c**) Top and bottom plates; (**d**) Plates with film electrodes; (**e**,**f**) Assembled sensor.

After printing, we rinsed the template thoroughly with isopropanol and ethanol to remove resin residue. Then, the template was exposed to ultraviolet (UV) light in a UV curing machine (Cure3D, MAKEX, Ningbo, China) for 1 h to ensure complete curing. After that, the template was placed in an air convection oven (PG-2J, ESPEC, Osaka, Japan) and heated to 120 °C for 3 h and cooled with the oven to eliminate the internal stress of the template and prevent deformation in the pouring process, as shown in Figure 6a.

Next, the PDMS elastomer and curing agent (Sylgard 184, Dow Corning, Midland, MI, USA) were mixed evenly at a ratio of 10:1 and vacuumed to remove bubbles. The 3D printed template was heated to 100 °C in the oven for 4 h after the mixture was cast. After that, the top and bottom plates were obtained by peeling off the cured PDMS from the template, as shown in Figure 6b.

Then oxygen plasma was used to clean the top and bottom plates to enhance the hydrophilicity of the PDMS surface by the low-pressure plasma system (V6-G, Pink, Wertheim, Germany). Subsequently, a mixture of PEDOT:PSS (P Jet 700 N, Clevios, Hanau, Germany) and PUD (Tekspro 7360, WANHUA, Yantai, China), with a ratio of 9: 1, was spread evenly by drop-casting to the top and bottom plate surfaces and cured completely at 70 °C for 30 min, as shown in Figure 6c. Then the carbon nanotube’s water-based coating (XFEC01, XFnano, Nanjing, China) was diluted to 1%wt and applied on the PEDOT:PSS/PUD films in the same way to achieve a double-layer conductive film, as shown in Figure 6d.

Finally, the top and bottom plates were tied together by the Kapton tape, and the fabricated pressure sensor is shown in Figure 6e,f.

**Figure 7:**
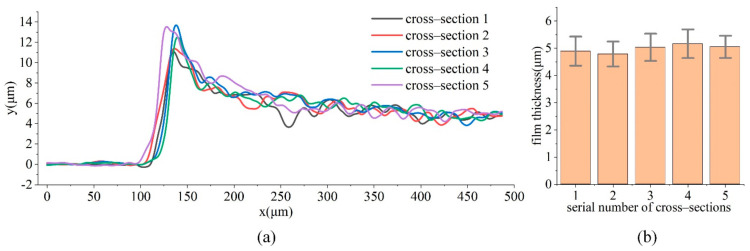
(**a**) The thickness of five cross-sections of the film; (**b**) Average thickness of the film between 350–450 μm in the *x*-axis position.

To measure the thickness of the film, the film step was prepared, and the film thickness was observed and measured with a 3D measuring laser microscope (LEXT OLS4100, OLYMPUS, Tokyo, Japan). Five cross-sections of the film step were measured, as shown in Figure 7a. Due to the unevenness of the film near the step (120–140 μm in the *x*-axis position), the average film thickness was calculated from the film thickness between 350 and 450 μm in the *x*-axis position, which was 4.99 ± 0.50 μm, as shown in Figure 7b.

## 3. Results

### 3.1. Experimental Setup

**Figure 8:**
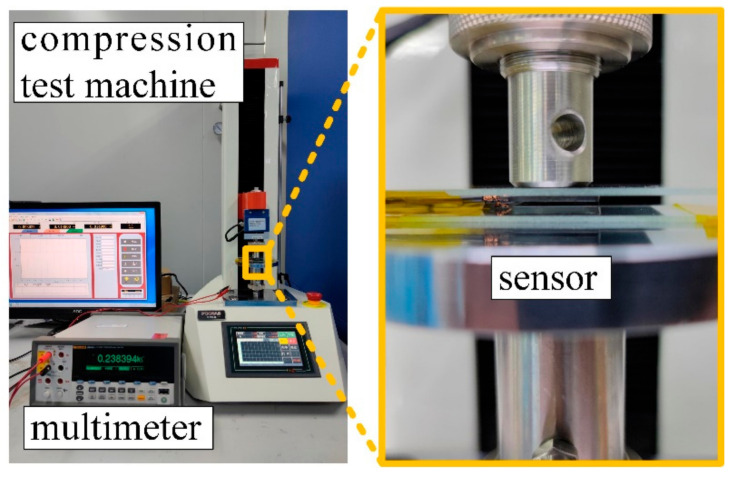
Experimental Setup.

The test bench mainly consisted of two parts, a compression test machine (PT-1198G, POOTAB, Dongguan, China) and a high-precision multimeter (8846A, Fluke, Everett, WA, USA) [35], as shown in Figure 8. The sensor was placed on the test machine plate, and the force was applied to the sensor by adjusting the displacement of the squeeze head. The resistance change of the sensor was read through the high-precision multimeter. The experimental setup was put in a 25 °C clean room, where the environmental impact can be minimized.

### 3.2. Static Test

**Figure 9:**
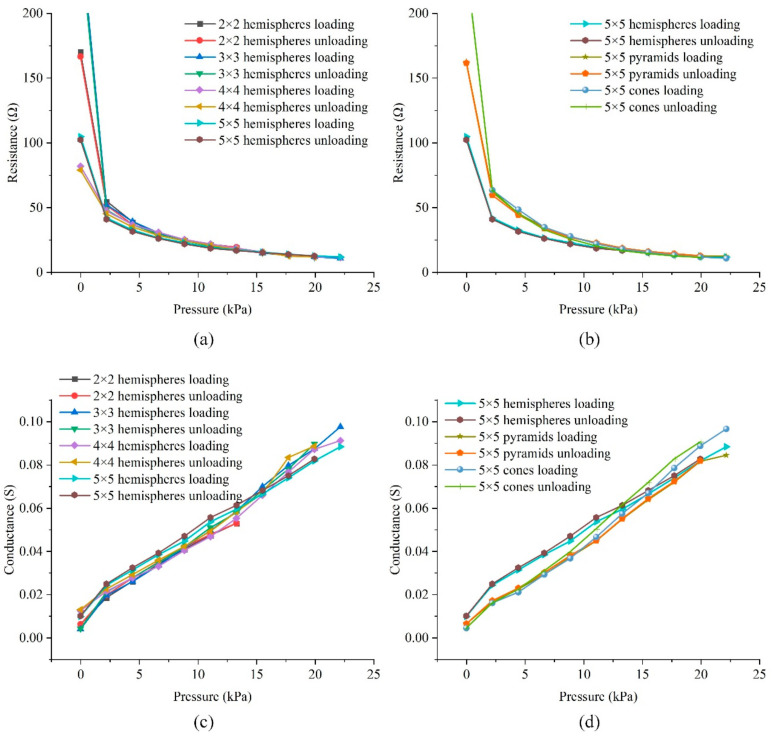
Sensors static test results: (**a**,**c**) Sensors with different hemispheres distribution densities; (**b**,**d**) Sensors with different structures.

In order to evaluate the static performance of sensors with different microstructure arrays, we tested the six types of sensors processed above. The test range of the sensor with 2 × 2 hemisphere structure was 0–13.3 kPa, and the range of the rest of the five sensors was 0–22.2 kPa. The force load started from 0 kPa, and increased by 2.22 kPa each time, with each step holding for 20 s. After reaching the maximum range, it decreased by 2.22 kPa each time. The pressure was also held for 20 s, and the resistance change of the whole period was read through the high-precision multimeter. As shown in Figure 9a,b, at low pressure ranges (0–4 kPa), the sensor resistance decreased sharply, but at high pressure (>4 kPa), since the microstructure deformation of the sensor saturated with the increase in pressure, the resistance decrease speed is significantly reduced. This result was consistent with the conclusion of the previous FEM result.

By converting resistance to conductance, as shown in Figure 9c,d, it can be seen that the conductance-pressure curves of the six sensors are almost linear and almost overlap, which is similar to the simulation conclusion. Taking a 5 × 5 hemisphere structure sensor as an example, it can be obtained by the calculation that the sensitivity of the sensor is 3.54 × 10^−3^ S/kPa in the range of 0–22.2 kPa. The maximum conductance difference between loading and unloading was 0.0023 S at 8.89 kPa, and the hysteresis was 1.41%FS. This was due to the viscoelasticity of the flexible film and the substrate, which could only be minimized but not completely eliminated. The largest deviation of the resistance appeared at 0 kPa; it was considered that the contact between the top and bottom plates was not sufficient, so the deviation was relatively large.

### 3.3. Temperature Test

**Figure 10:**
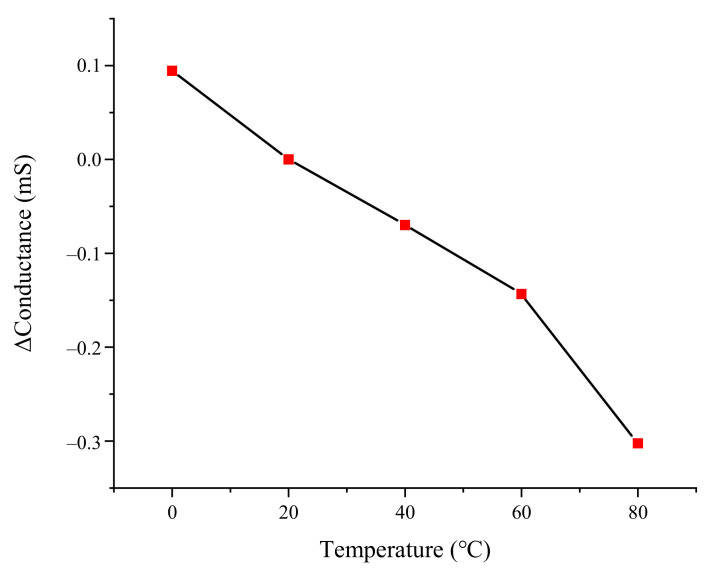
Thermal zero drift of the sensor.

The temperature test of the pressure sensor with 5 × 5 hemispheres was conducted within a temperature range from 0 to 80 °C in the air convection oven. The output data is shown in Figure 10, and the maximum change of the output conductance was −0.302 mS at 80 °C. The zero-temperature coefficient was calculated, which was −0.0064%FS/°C. It might be caused by the thermal expansion of the PDMS substrate and the PEDOT:PSS/PUD film electrodes.

### 3.4. Durability Test

**Figure 11:**
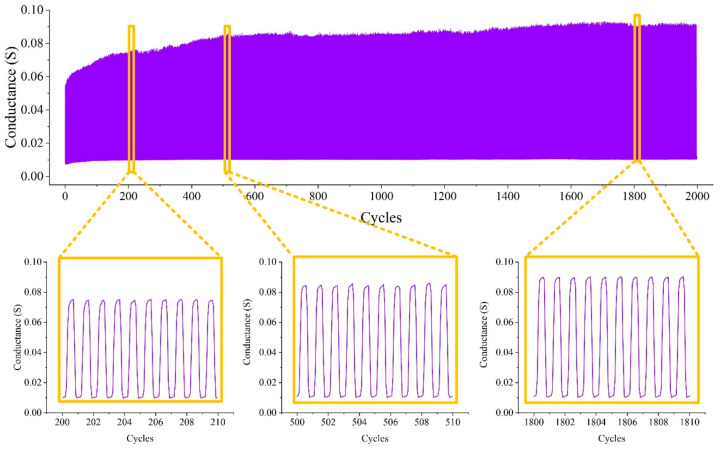
Durability test results.

By controlling the movement of the compression test machine, the sensor with 5 × 5 hemispheres had undergone 2000 loading/unloading tests, and each loading/unloading process took 2 s. The data was read by the high-precision multimeter, and the results are shown in Figure 11.

Before 500 cycles, there was an obvious increase in conductance from 0.06 to 0.082 S during loading. The conductance of unloading remained stable at about 0.01 S during the whole test. Judging from the magnified images of 500–510 cycles and 1800–1810 cycles, the conductance change curve of each load/unload cycle was almost the same, which also reflected the good repeatability of the sensor. It can be seen from the rise and fall speed of the resistance that the response speed of the sensor was very fast.

### 3.5. Effect of Test on Electrode Films

**Figure 12:**
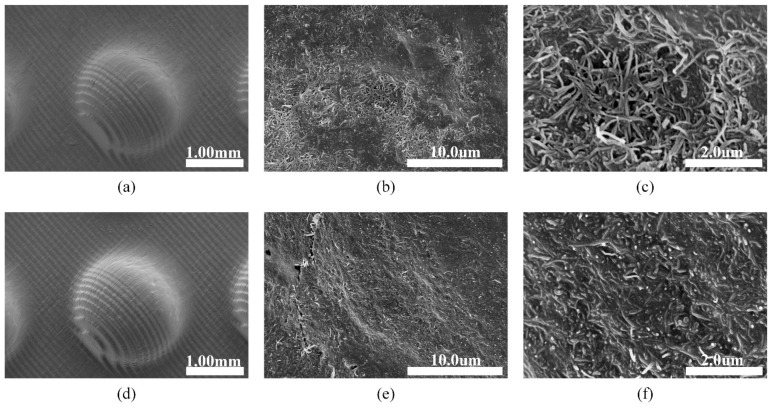
SEM images of sensors before (**a**–**c**) and after (**d**–**f**) the test: (**a**,**d**) Surface of the structure; (**b**,**c**,**e**,**f**) Top of the hemisphere under higher magnification.

The surfaces of the sensor before and after the test were observed using the scanning electron microscope (SEM) (Hitachi, SU8010, Japan), as shown in Figure 12. Figure 12a–c was the sensor image before the test. Under low magnification, stripes could be seen on the sensor surface, which was due to the 3D printing mold formed layer by layer, and the layer thickness was set to 50 μm during printing. Under high magnification, the distribution of carbon tubes on the surface of the sensor could be clearly seen. It can be seen in the electron microscope image of the sensor after the durability test that although the electrode film seemed intact at low magnification (Figure 12d), slight cracks on the electrode film can be found at high magnification (Figure 12e). At higher magnification, the loose CNTs have been compacted and embedded in the lower PEDOT:PSS/PUD hybrid film (Figure 12f).

## 4. Conclusions

In this work, we discussed the influence of protrusions with different morphologies and different density distributions on flexible pressure sensors performance and developed a new method of manufacturing sensors based on 3D printing technology. The elastic top and bottom plates of the sensor with micro-structure protrusions were made of PDMS, and the surfaces of the plates were coated with a conductive film, which was made from a mixture of PEDOT:PSS and PUD, and a layer of CNTs covered it. The top and bottom plates were obtained by replicating a 3D printed template. Compared with the previous templates obtained by the silicon process, 3D printing has the advantages of time-saving, low cost and being able to process unique shapes. By using FEM simulation, three sensors with different types of microstructures, including pyramid, cone and hemisphere, were analyzed, and the results show that the sensor with the hemispherical structure had the best performance. Furthermore, sensors with different hemisphere density distributions had similar responses. The difference was that the range of the sensor increased as the hemisphere density increased. All sensors were fabricated and tested. The sensor with 5 × 5 hemispheres had a sensitivity of 3.54 × 10^−3^ S/kPa and hysteresis of 1.41%FS in the range of 0–22.2 kPa. The zero-temperature coefficient was −0.0064%FS/°C. The durability test was carried out for 2000 cycles, and the sensor remained stable during the test. These results prove that 3D printing has great potential in the field of sensor manufacturing. In future research, higher precision 3D printers and suiTable 3D printing materials will optimize the processing and improve the performance of the sensor.

## Data Availability

Data sharing not applicable.
